# Utilization of Deep Eutectic Solvents to Reduce the Release of Hazardous Gases to the Atmosphere: A Critical Review

**DOI:** 10.3390/molecules26010075

**Published:** 2020-12-26

**Authors:** Irfan Wazeer, Mohamed K. Hadj-Kali, Inas M. Al-Nashef

**Affiliations:** 1 Chemical Engineering Department, King Saud University, P.O. Box 800, Riyadh 11421, Saudi Arabia; iwazeer@ksu.edu.sa; 2 Department of Chemical Engineering, Masdar Institute, Khalifa University of Science and Technology, P.O. Box 54224, Abu Dhabi, UAE; enas.nashef@ku.ac.ae

**Keywords:** deep eutectic solvents, climate change, human health, CO_2_ capture, toxic gases, desulfurization, denitrogenation

## Abstract

The release of certain gases to the atmosphere is controlled in many countries owing to their negative impact on the environment and human health. These gases include carbon dioxide (CO_2_), sulfur oxides (SOx), nitrogen oxides (NOx), hydrogen sulfide (H_2_S) and ammonia (NH_3_). Considering the major contribution of greenhouse gases to global warming and climate change, mitigation of these gases is one of the world’s primary challenges. Nevertheless, the commercial processes used to capture these gases suffer from several drawbacks, including the use of volatile solvents, generation of hazardous byproducts, and high-energy demand. Research in green chemistry has resulted in the synthesis of potentially green solvents that are non-toxic, efficient, and environmentally friendly. Deep eutectic solvents (DESs) are novel solvents that upon wise choice of their constituents can be green and tunable with high biocompatibility, high degradability, and low cost. Consequently, the capture of toxic gases by DESs is promising and environmentally friendly and has attracted much attention during the last decade. Here, we review recent results on capture of these gases using different types of DESs. The effect of different parameters, such as chemical structure, molar ratio, temperature, and pressure, on capture efficiency is discussed.

## 1. Introduction

Climate change is an exceedingly critical environmental challenge, and its mitigation and remediation have gained widespread attention. Many countries around the world, have instituted laws and regulations to maintain environmental air quality and control the emission of pollutants that harm human health and affect the atmosphere [1]. Six major air contaminants have been identified based on National Ambient Air Quality Standards, including ozone, particulate matter, carbon monoxide, sulfur oxides (SOx), nitrogen oxides (NOx), and lead. Long-term exposure to these contaminants has been shown to have a harmful impact on human wellness over decades, evincing in a broad range of problems, including higher infant mortality rates and inherited respiratory diseases [2]. SOx and NOx, in particular, have serious effects on multiple human organs, damaging the nervous, respiratory, gastrointestinal, and cardiovascular systems to a degree that has been proven to be lethal. When gases such as SOx and NOx are transformed via chemical reactions, a portion of particulate matter is produced in the air. In the presence of sunlight, ozone is formed by chemical reactions between volatile organic compounds (VOCs) and NOx. Both SOx and NOx have harmful effects, in addition to their severe impacts on human health and climate, because they generate other air pollutants. A fossil-fuel driven plant releases approximately 80% of the NOx and 70% of the SOx present in the surrounding atmosphere, making oxide mitigation and elimination a critical task in automotive and industrial processes [3].

In the petrochemical industry, fuel is a primary source of pollutants in the environment, as it is rich in aromatics, nitrogen and sulfur-containing aromatic substances that are burned to create harmful contaminants. Thus, both the climate and human health can be influenced by the composition of fossil fuel oils [4]. Strict environmental standards to eliminate aromatics and sulfur- and nitrogen-containing fuel oil content have been implemented around the world to improve air quality. In short, harmful emissions are to be restricted by the generation of clean-burn fuel oils. In addition, the aromatic products of sulfur and nitrogen are troublesome in the refining of oil and gas because they are the sources of catalytic toxicity or deactivation, degradation and gum formation. High levels of aromatic content have been shown to affect the quality of the fuel; it is therefore important that aromatic compounds be separated from aliphatic ones [5]. Currently, hydrodesulfurization (HDS) is a proven desulfurization process that is widely used in the industry [6]. Aliphatic hydrocarbon sulfur content can be efficiently eliminated by HDS. However, because of their broad steric hindrance, it is difficult for polycyclic organic sulfides such as thiophene, benzothiophene, dibenzothiophene, and their derivatives to attain deep removal. Even if deep desulfurization can be accomplished, operating conditions are excessively harsh, resulting in a significant increase in the cost of desulfurization. Inhibition of the HDS process by the presence of nitrogen-containing compounds that poison the catalysts has also stimulated the need for denitrogenation. Hydrodenitrogenation is the most prominent denitrogenation process in the industry, but it involves harsh working conditions, and significant hydrogen consumption and capital costs. Furthermore, it is difficult to reach high denitrogenation efficiency [7]. The nitrogen content limit in diesel fuel has been regulated in many countries since 2011, and the allowed concentration was lowered from 70 ppm to <0.10 ppm. Comparably, in most countries, the sulfur content has been set to as low as 10 ppm (on an annual average basis) [8].

The rise in global temperatures constitutes an emergent risk to the earth, with an estimated 2 °C rise predicted by the end of this century [9]. The phenomenon of a gradual rise of average global temperature is known as the greenhouse effect and can be attributed to a group of greenhouse gases, of which carbon dioxide (CO_2_) is the most dominant [10]. A direct cause of climate change is the steady annual growth of CO_2_, which is responsible for over 70% of the world’s estimated greenhouse gases. Additionally, CO_2_ has direct adverse health consequences on human health, with signs varying from acute breathlessness to lack of cognitive capacity, based on the degree and length of exposure to CO_2_. Such symptoms can be acute or chronic and can have a detrimental impact on human health if excessive CO_2_ exists in indoor and outdoor air [11].

**Figure 1:**
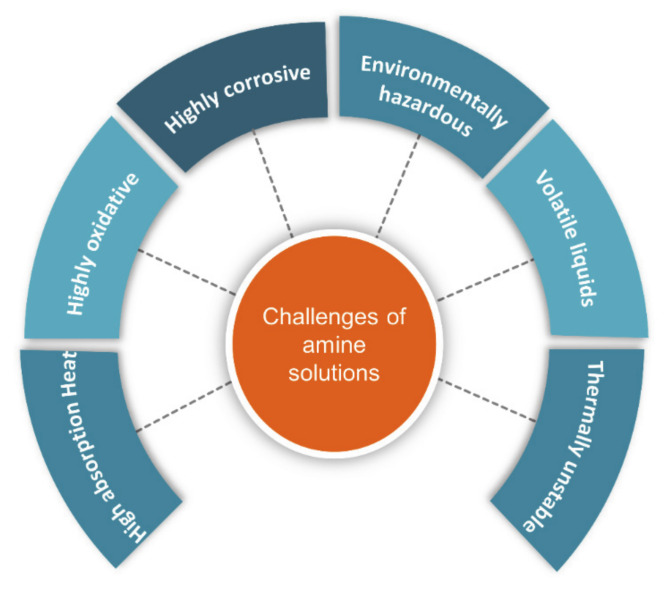
Major challenges of using amine solutions [15,16,17].

Various processes have been developed for the removal of toxic gases, including adsorption, absorption, and membrane separation [12,13]. Amine scrubbing is the most prominent among chemical absorption methods [14]. The most widely used amine solvents for CO_2_ removal are monoethanolamine (MEA), diethanolamine (DEA), triethanolamine (TEA), diglycolamine (DGA), and methyldiethanolamine (MDEA). However, major downsides of amine scrubbing methods include the degradation of amines, the corrosion caused by the components produced during amine reactions with CO_2_ and high regeneration energy (Figure 1) [15,16,17]. Other good CO_2_ absorbents include caustic solvents such as calcium hydroxide, potassium hydroxide, and sodium hydroxide [18]. Unfortunately, the regeneration of solvents remains a big concern. Carbonate solutions have also been employed for the absorption of acidic gases. While carbonate solutions have low volatility and result in a lower corrosion rate, they have major drawbacks in terms of lower absorption rates in comparison with caustic and amine solutions [19]. Compared to caustic and amine solutions, amino acid salt solutions have the benefit of improved regeneration owing to their lower volatility. However, the high molecular weight of amino acid salt solutions is a major drawback and increases the capital expenses of the absorber [20]. Owing to its simplicity of design, energy efficiency, and ease of scale-up, the use of membrane technology to capture CO_2_ has seen rapid growth in popularity. Unfortunately, most membranes require regular replacement because they do not last long in practical industrial environments [21].

In recent decades, medicinal, chemical, and industrial processes have widely utilized solvents accounting for nearly 30% of emissions of VOCs and 60% of industrial emissions worldwide [22]. Green processes in all areas of chemistry and engineering have drawn significant attention over the past 20 years, representing an increasing desire to minimize the usage of hazardous and unsafe chemicals and to raise understanding of environmental concerns. Green chemistry and engineering is also responsible for limiting or eliminating the usage of harmful and hazardous chemicals and for designing ecologically sustainable chemical procedures [23]. Solvents comprise a significant field of research in green chemistry with most of the traditional chemicals being toxic and flammable. Therefore, researchers have established many safer alternatives, such as water-based or solvent-free systems, ionic liquids (ILs), and supercritical fluids (SCF), with the solvent-free system representing the best-case scenario [24,25]. However, the use of solvents cannot always be eliminated because of their pivotal role in heat and mass transfer, and dissolution and separation operations [26]. For example, water, the world’s most abundant compound, has already been used in a number of chemical processes as a solvent. Even so, the drawbacks of using water as a solvent include the negligible solubility of various organic compounds and the possibility of contamination, with a further downside posed by the high vaporization energy of this solvent. Another solution is to substitute typically organic solvents with SCFs; that are chemically stable, easy to handle, and safe. However, owing to their non-polar existence, SCFs still have some drawbacks, e.g., CO_2_ has minimal dissolving capacity for polar solutes [27].

Since the early 2000s, ILs, a form of newly synthesized solvents, have garnered a great deal of attention from the research community in multidisciplinary fields. ILs are molten salts, composed of ions, with a melting point below 100 °C [28,29]. The main aspects of ILs over conventional organic solvents are their minimal vapor pressure, good thermal characteristics, wide liquid range, miscibility, solubility range, and chemical reaction suitability. Nevertheless, studies indicated that the ‘green’ characteristics of ILs are at least questionable because of the known drawbacks of these solvents; for example, high preparation costs, high viscosity, equivalent or higher toxicity, and poor biodegradability compared to organic solvents [30,31].

Deep eutectic solvents (DESs) have become attractive replacements for traditional solvents and ILs in order to tackle the high levels of toxicity and costs of ILs. However, the definition of DESs remains controversial, and different definitions do not discriminate DESs from other mixtures, as all mixtures of immiscible solids are eutectic and various compounds may form hydrogen bonds when mixed [32]. The concept of DESs as a new class of sustainable solvent was first coined by Abbott et al., and identified as solvents with melting points significantly lower than those of individual components when combined in a proper molar ratio [33]. DESs consist of large, nonsymmetric ions with low lattice energy and thus low melting points and are usually produced by the mixing of a metal salt or hydrogen bond donor (HBD) and a quaternary ammonium salt. The delocalization of charges between HBD and, for example, a halide ion via hydrogen bonding allows the melting points of the mixture to decrease in comparison with the melting points of the individual constituents. Recently, Martins et al. [34] defined DES as a blend of two or more components with a eutectic temperature that is lower than that of an ideal liquid mixture, with substantial negative deviations from ideality (∆T_2_ > 0). ∆T_2_ indicates the depression in temperature, which is the difference between the ideal and real eutectic point. Furthermore, these authors indicated that it is critical that, irrespective of the composition of the mixture, the temperature depression contributes to the liquid mixture at the operating temperature.

**Figure 2:**
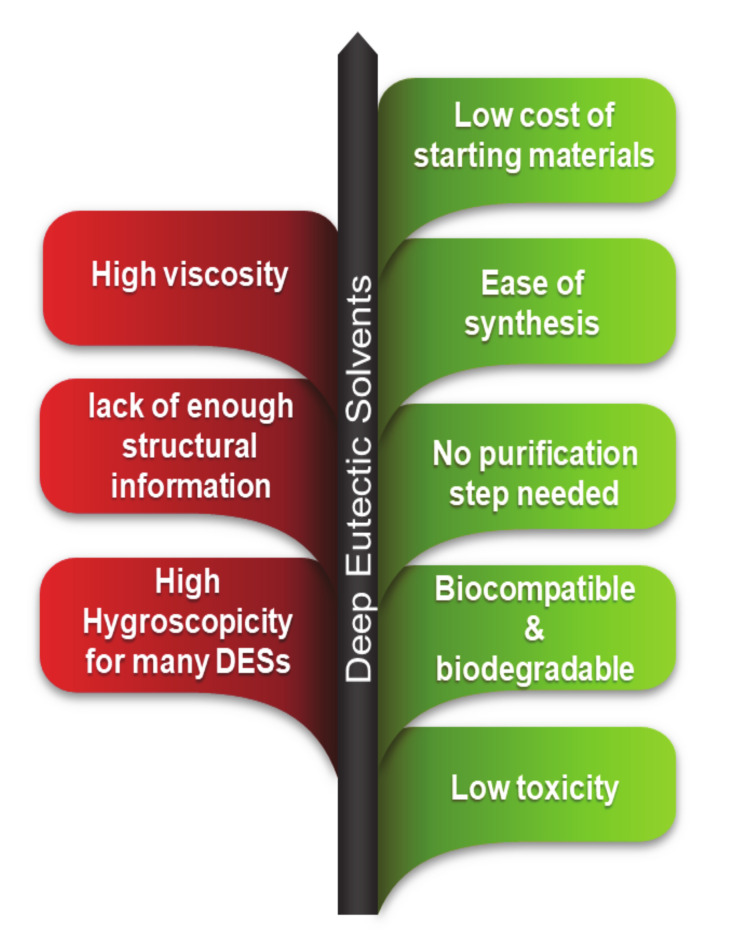
Merits and limitations of DESs as potential solvents [22,48,50].

Although DESs and traditional ILs have different chemical properties, they have similar physical properties, especially their capacity to be tailored to a specific form of chemistry as tunable solvents. They also possess low vapor pressure, a relatively broad liquid-range, and non-flammability [35]. DESs have many benefits over conventional ILs, such as easy preparation and a convenient supply of comparatively cheap materials (the components themselves are toxicologically well characterized, so they can be conveniently transported for manufacturing on a broad scale); they are, however, less chemically inert in general. The preparation of DESs requires the simple mixing of two or more compounds, usually with moderate heating. With respect to traditional ILs (e.g., imidazolium-based liquids), this yields a relatively low preparation cost and enables large-scale applications. The toxicity of ammonium-based DESs toward bacterial and eukaryotic cells was first investigated by Hayyan et al. [36] and no inhibition of bacterial growth was observed. However, DESs based on phosphonium salts were found to be cytotoxic under the same conditions [37]. Mao et al. [38] found that the toxicity of ChCl-based DESs toward *Arthrobacter simplex* was lower than that of the individual components. The toxicity and biodegradability of DESs toward various organisms (such as, bacterium, *Escherichia coli*) was assessed by Wen et al. [39]. The investigated DESs were toxic to bacteria at concentrations above 75 μM and inhibited bacterial growth by the DESs was much more than that of their individual components. Radosevic et al. [40] investigated the toxicity of three ammonium-based DESs, utilizing fish and human cell lines to measure the in vitro toxicity. All examined DESs were rated as readily biodegradable based on their high degree of mineralization. Many researchers have indicated that DESs has minimal or negligible volatility relative to traditional organic solvents. However, recently, Chen et al. [41] reported the volatilization of ChCl:*N*-methylacetamide (1:6) DES even at room temperature and pressure. Another study conducted by the same group [42] found that the polyethylene glycol (PEG)-based DESs showed volatilization under the same conditions. It is obvious that a better understanding of DESs volatility and toxicity needs more research before these solvents can really be claimed to be less-volatile, biodegradable and non-toxic. A few merits and disadvantages of DESs are presented in Figure 2.

DESs play a significant role in the solubility of gases and liquid-liquid extraction (LLE). Our group [43] compared the performance of classical amine solvents (widely used in the industry for this purpose) and the amine-based DESs for CO_2_ capture. The results showed that only 10% of the amine reacted with the CO_2_ while for the aqueous solution of MEA, all of the amine reacted with the CO_2_. Hence, if only 10% of the amine in the DES reacts with CO_2_, this means that the energy needed for desorption is much less than that in amine aqueous solution. In another application related to the use of DESs for separating aromatics from aliphatics, it was noticed that when sulfolane-based DES was used as liquid-liquid extractant, no DES was found in the raffinate layer [44]. While when pure sulfolane was used, its concentration in that layer reached 20 wt%; causing solvent loss and requiring additional purification steps. The same behavior could be predicted for desulfurization and denitrification exposed in this review.

In 2015, García et al. [45] presented a review on the applications of DESs for gas solubility, with special attention to CO_2_ capture. Some other reviews involving the capture of CO_2_ via DESs have also been published [46,47,48]. In this work, we present a critical review for the use of different types of DESs in capturing CO_2_. Because some reviews about this topic have already been published, we limit the review to the articles published starting from 2015; however, we give more attention to articles published recently. Chen et al. [49] provided a review on the capture of toxic gases by DESs. However, the review about the desulfurization and denitrification of fuels using DESs was not presented. In this study, the use of DESs in the absorption of other hazardous gases, e.g., SOx, NOx, NH_3_, etc. will be discussed in detail. Furthermore, we will also present in dept review about the desulfurization/denitrification of fuels via DESs. Finally, challenges, opportunities, and perspective of the commercial use of DESs will be discussed.

The main topics of this study are as follows:

- CO_2_ capture
- Capture of acidic gases
- Selectivity of DESs in capturing gases
- Desulfurization and denitrification of fuels

## 2. CO_2_ Capture

Chemical absorption, especially amine-based processes, is one of the preferred options for capturing CO_2_. However, this process involves some major drawbacks, including the high cost of this technology associated with extensive energy penalties, solvent degradation, and high corrosion [51,52]. The cost of CO_2_ separation using amine-based technology is in the range of US $50 to US $100 per ton of carbon, which is very high for most of the applications [53]. DESs have become desirable solvents for various gas technology applications due to their beneficial characteristics, such as biodegradability, good thermal and chemical stability, non-flammability, high solvation capability, low-cost, and ease of preparation. This article provides a comprehensive review of the potential applications of DESs for CO_2_ capture based on recent studies. Various studies concerning the capture of CO_2_ via DESs have already been published; therefore, in this article, we have considered studies published since 2015. Choline chloride (ChCl)/urea (1:2) is one of the most widely investigated DES for CO_2_ capture. The solubility of CO_2_ was first measured in ChCl/urea (1:2) DES at 313.15, 323.15, and 333.15 K and at pressures up to 13 MPa [54]. Many factors influence the solubility of CO_2_ in DESs, including pressure, temperature, the type of hydrogen bond acceptor (HBA) or HBD, the HBA:HBD molar ratio, viscosity, and the water content of DESs. Table 1 displays the solubility data of CO_2_ in various DESs at different temperatures and pressures. We have only included DES systems with CO_2_ solubility higher than 0.1 mol CO_2_ kg^−1^ solvent.

**Figure 3:**
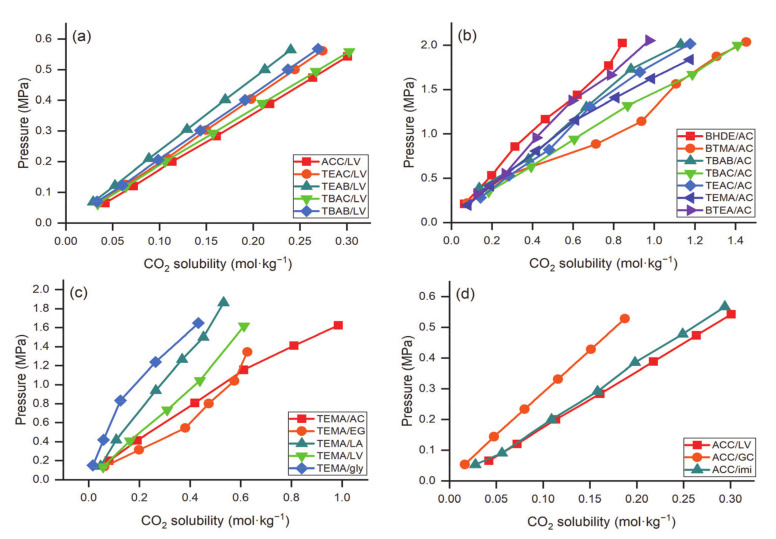
Effect of (**a**) HBA of LV-based DESs on CO_2_ solubility at 303.15 K, (**b**) HBA of AC-based DESs on CO_2_ solubility at 298.15 K, (**c**) HBD of TEMA-based DESs on CO_2_ solubility at 298.15 K, and (**d**) HBD of ACC-based DESs on CO_2_ solubility at 303.15 K, data are extracted from [56,57,61].

The unit for the comparison of CO_2_ capture is mol·kg^−1^, except as otherwise mentioned. Deng et al. [58] examined the effect of the HBA of DESs on CO_2_ solubility in five LV-based DESs as illustrated in Figure 3a. All DESs were prepared at 1:3 HBA to HBD molar ratio. At fixed temperature (i.e., 303.15 K) and pressure (~0.55 MPa), ACC/LV and TBAC/LV DESs demonstrated the highest CO_2_ absorption capacity (around 0.3), while TEAB/LV had the lowest value (0.24). Sarmad et al. [61] also investigated CO_2_ capture by various DESs with the HBD as acetic acid (AC). The effect of HBA in the DESs for CO_2_ capture was ordered as follows: BTMA (1.45) > TBAC (1.41) > TEAC (1.18) ≈ TEMA (1.18) > TBAB (1.13) > BTEA (0.97) > BHDE (0.84) at a 1:2 molar ratio (HBA:HBD), 298.15 K, and pressure around 2 MPa, as shown in Figure 3b. The authors also examined the effect of the HBD; for example, TEMA-based DESs were prepared by mixing TEMA as the HBA with five different HBDs including AC, ethylene glycol (EG), glycerol (gly), LV and lactic acid (LA) at a 1:2 molar ratio (Figure 3c). The TEMA-based systems followed the order: TEMA/AC (0.61) > TEMA/EG (0.57) > TEMA/LV (0.44) > TEMA/LA (0.37) > TEMA/gly (0.26) at 298.15 K and pressure around 1 MPa. These variations were due to the interactions between CO_2_ and functional groups in the HBD. The intermolecular hydrogen bonds are stronger in LA than in AC or LV because of the proximity of the hydroxyl group to the carboxylic group. Hence, it is not easy to break intermolecular hydrogen bonds for contact with CO_2_. Furthermore, acetic acid has the weakest intermolecular hydrogen interactions; therefore, acetic acid molecules can readily interact with CO_2_, yielding higher CO_2_ solubility compared to other DESs. For acetic acid-based DESs, the solubility of CO_2_ increases with increasing alkyl chain length of the HBA. For instance, when the alkyl chain length increased from ethyl to butyl (i.e., from tetraethylammonium to tetrabutylammonium), the solubility of CO_2_ increased from 1.177 to 1.411 mol·kg^−1^. Similar behavior was reported by Zubeir et al. [62]; i.e., the solubility of CO_2_ increased by increasing the alkyl chain length from methyltrioctyl- to tetraoctylammonium. Moreover, an increase in carbon atoms in the HBD alkyl chain increases CO_2_ solubility. This can be attributed to the increase in free volume with increasing alkyl chain length, resulting in higher CO_2_ solubility [64]. Li et al. [65] synthesized a series of DESs by mixing different ammonium salts such as HBA and MEA, DEA, MDEA, and TEA as HBDs for CO_2_ absorption. The solubility of CO_2_ followed the following trend: ChCl ≈ TMAC > TEAC > TEAB > TBAC > TBAB, while for HBD, the order is MEA > DEA > MDEA > TEA. MDEA and TEA showed low CO_2_ absorption because of the absence of hydrogen on the nitrogen atoms. The solubility of CO_2_ using ChCl and TMAC was almost the same because both salts have similar chemical structures. In Figure 3d, the effect of HBD of ACC-based DESs was investigated at 303.15 K. ACC-based DESs were prepared by mixing with LV, guaiacol (GC), and imidazole (imi) at a 1:3 molar ratio. The order for CO_2_ solubility was ACC/LV (0.3) > ACC/imi (0.29) > ACC/GC (0.18).

**Figure 4:**
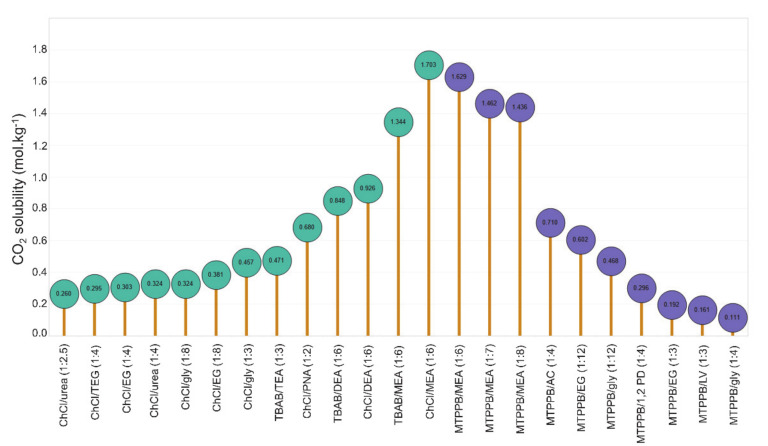
CO_2_ solubility in ammonium and phosphonium-based DESs at around 1 MPa, and 298.15 K. Data are taken from [43,61].

The nature/type of salt of DESs also plays a crucial role in CO_2_ capture. Deng et al. [58] used five ammonium salts (ACC, TEAB, TEAC, TBAB, and TBAC) to prepare DESs for CO_2_ capture. For ammonium salts, DESs with larger cations showed higher CO_2_ solubility, and the cations of the salts dominated the absorption capacity for CO_2_ capture. The performance of various ammonium- and phosphonium-based DESs for CO_2_ capture is compared in Figure 4 [43,61]. Both types of DESs with MEA as the HBD resulted in higher CO_2_ solubility than the DEA- and TEA-based DESs. The solubility of CO_2_ increased by increasing the HBA:HBD molar ratio for ChCl/EA DESs; however, the opposite was true for MTPPB:MEA DES [66].

**Figure 5:**
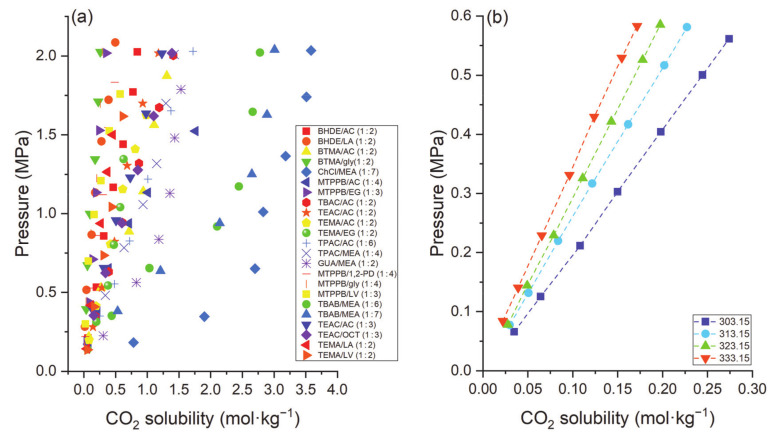
Effect of pressure on (**a**) various DESs at 298.15 K, and (**b**) TEAC/LV (1:3) DES at different temperatures. Data are extracted from [58,61].

In general, the solubility of CO_2_ in DESs increases with decreasing temperature and increasing pressure. Sarmad et al. [61] investigated the effect of pressure on the CO_2_ capture via various DESs, as shown in Figure 5. As expected, the solubility of CO_2_ in the DESs increased with increasing pressure and decreasing temperature for the systems examined. The decrease in solubility with increasing temperature can be understood using the concept of the kinetic energy of the gas molecules: as it increases with increasing temperature, causing breakage of intermolecular bonds between the gas molecules that are formed within the solute, and these have a higher tendency to escape from the solution.

Usually, the solubility of CO_2_ in the DESs follows Henry’s Law (Equation (1)), i.e., the solubility of CO_2_ is proportional to its partial pressure. Deng et al. [58] also investigated the effect of pressure on CO_2_ solubility in five LV-based DESs. They found that the solubility of CO_2_ in DESs is proportional to the gas phase’s equilibrium pressure, indicating that the CO_2_ absorption via DES is a physical phenomenon. Figure 5a depicts the CO_2_ solubility as a function of pressure in various DESs. It is evident from the figure that the solubility increases with increasing pressure for all the DESs. Figure 5b shows the effect of pressure on TEAC/LV (1:3) at different temperatures. Solubility trends have shown that the CO_2_ absorption capacity increases with decreasing temperature and increasing pressure.

The degree of gas solubility in a solvent is often assessed using Henry’s law constant (*k_H_*) [67]:

(1) $$k_{H}=\;\underset{x_{i}\to 0}{\lim}\left(\frac{f_{i}}{x_{i}}\right)$$

where *x_i_* is the mole fraction of gas in the solution, and *f_i_* is the gas fugacity in vapor phase.

**Figure 6:**
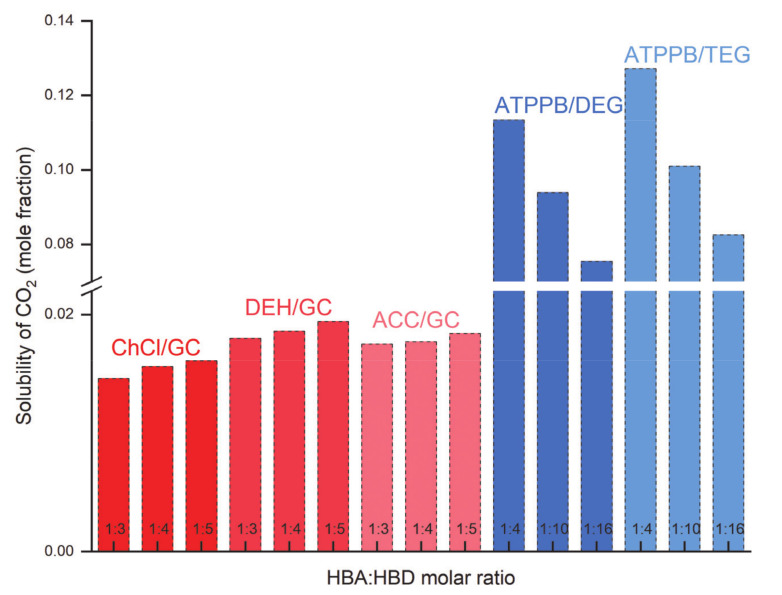
Effect of the molar ratio of DESs on the solubility of CO_2_ at 303.15 K and under 0.5 MPa. Data are extracted from [57,60].

Liu et al. [57] reported that the solubility of CO_2_ increases with an increase in the mole fraction of ChCl/GC, DEH/GC, and ACC/GC from 1:3 to 1:5 at a fixed pressure and temperature, indicating that GC plays a major role in the solubility of CO_2_ in DESs. For example, the solubility of CO_2_ using ChCl/GC increased from 0.171 to 0.188 when molar ratio was changed from 1:3 to 1:5. Furthermore, the effect of the molar ratio of DESs on Henry’s law constant of CO_2_ absorption was also obvious. Among GC-based DESs, DEH/GC (1:5) showed the lowest Henry’s law constant, i.e., higher CO_2_ solubility at a fixed temperature and pressure. The effect of the molar ratio of different amine-based DESs on the solubility of CO_2_ was also investigated [66]. The solubility of CO_2_ increased with a decrease in the molar ratio of ChCl/MEA and ChCl/DEA DESs from 1:6 to 1:10, indicating that addition of MEA or DEA can increase both the chemical and physical absorption of CO_2_. However, Ghaedi et al. [60] found that by increasing the molar ratio of phosphonium-based DESs including ATPPB/diethylene glycol (DEG), and ATPPB/triethylene glycol (TEG) from 1:4 to 1:16, the solubility of CO_2_ decreased as shown in Figure 6. This result demonstrated that ATPPB plays an important role in CO_2_ capture. At the same time, glycols play a minor role. Ren and co-workers [68] explored the effect of L-arginine/gly molar ratios (1:5, 1:6, and 1:7) on the solubility of CO_2_.

Many DESs are highly hygroscopic and tend to absorb water easily [69]. Ren et al. [68] exploited the hydrophilic nature of DESs for CO_2_ capture. DESs with varying water content have been tested for CO_2_ capture to explore the effect of water content on CO_2_ solubility. They found that the efficiency of CO_2_ capture could be increased by adding a small amount of water to the DES. Ma et al. [70] investigated the effect of water on CO_2_ solubility using glycerol-based DESs. Most of the glycerol-based DESs have high viscosity; however, for some DESs, viscosity changes drastically when a small amount of water is added [71]. For instance, the viscosity of BTMA/gly (1:2) DES was reduced from 716 to 20 mPa·s after adding a small quantity of water (0.11 mole fraction).

Furthermore, CO_2_ absorption increased by 25% using BTMA/gly (1:2) with 0.11 mole fraction of water. However, the solubility of CO_2_ was reduced by further increasing the water content owing to the low solubility of CO_2_ in water. Trivedi et al. [72] examined the absorption of CO_2_ in the presence of varying water content (5–20 wt%) with monoethanolamine hydrochloride /ethylenediamine (1:3) DES. With an increase in water content, the initial uptake kinetics were improved, i.e., when the water quantity increased from 0 to 20 wt%, CO_2_ uptake increased from 25.2 to 28.1 wt%.

## 3. Capture of Acidic Gases

Nitric oxide (NO), nitrogen oxide (NO_2_), ammonia (NH_3_), sulfur dioxide (SO_2_), and hydrogen sulfide (H_2_S) are considered toxic industrial gases [49]. The burning of coal mainly produces NO_2_ and NO. Large amounts of these oxides (NO and NO_2_) can cause acid rain, acid mist, destruction of ozone, and harm to human health. NH_3_ is often generated from waste gas during the synthesis of ammonia, which causes air pollution and rhinitis/pharyngitis and facilitates the formation of particulate matter. During the combustion of fossil fuels from industrial waste gas or volcanic eruptions, toxic SO_2_ is produced. Air pollution, human cancer, and acid rain result from the release of large quantities of SO_2_ [73]. H_2_S is another acidic gas produced from natural gas treatment, decomposition of bacteria, and industrial refineries. H_2_S has high toxicity and corrosiveness. Due to the high risk of these acidic gases (i.e., SO_2_, NH_3_, H_2_S, NO_2,_ and NO), high-performance, low-cost, and highly sustainable processes are required to capture them. It is clear that toxic gas capture can enhance air quality, reduce air pollution, and preserve the ozone layer. In addition, the captured gas can be utilized in other processes. Hence, it is imperative to have green routes to capture these toxic gases with better efficiency and selectivity.

The solubility of these gases in the solvent is an essential factor for the absorption of these gases. The solubility data for oxide-based acidic gases in various DESs and ILs are shown in Table 2.

### 3.1. Oxide-Based Acid Gases

#### 3.1.1. SO_2_ Capture

**Figure 7:**
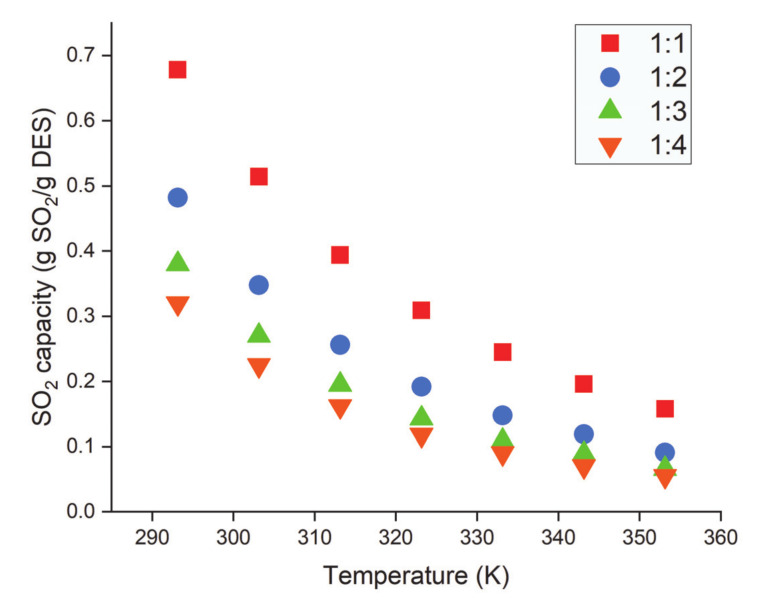
Effect of temperature and molar ratio on SO_2_ capture capacity by ChCl/gly. Data are extracted from [80].

Yang et al. [80] investigated the solubility of SO_2_ in ChCl/gly DES with different molar ratios. Figure 7 shows the effect of ChCl/gly DES molar ratio on the solubility of SO_2_ at different temperatures. The highest SO_2_ solubility (0.678 g SO_2_/g DES) using ChCl/gly was achieved at 1:1 molar ratio, 0.1 MPa, and 293.15 K. An increase in the HBD molar ratio from 1:1 to 1:4 at fixed pressure and temperature reduced the SO_2_ absorption capacity. For instance, at 293.2 K and 0.1 MPa, the solubility of SO_2_ was reduced from 0.678 to 0.320 (g SO_2_/g DES). The effect of a mole fraction of ChCl/gly DES on Henry’s law constant of SO_2_ absorption was also evident. Henry’s law constant increased with decreasing concentrations of ChCl in the DES. The same group [104] investigated the solubility of SO_2_ in another DES formed by combining EMIMC with EG under different operating conditions. The absorption capacity of SO_2_ increased (0.82, 1.03 to 1.15 g SO_2_/g DES) with an increase in the ratio of EMIMC in EMIMC:EG DES (from 1:2 to 1:1 and 2:1). The same group prepared EMIMC-based DESs by mixing EMIMC with either TEG or succinonitrile (SNT) to investigate the SO_2_ absorption capacity [105]. The SO_2_ absorption capacity increased with an increase in the molar ratio of EMIMC in the EMIMC/TEG DES. The highest capacity of 1.25 g SO_2_/g DES was achieved in the EMIMC/TEG at a 6:1 molar ratio.

It is interesting to note that in some cases, increasing the molar ratio of HBD increased the SO_2_ solubility. In contrast, in other cases, the solubility was increased by increasing the molar ratio of HBA. For example, the solubility of SO_2_ increased when the molar ratio of ChCl or EMIMC in ChCl/phenol, ChCl/gly, EMIMC/EG, EMIMC/TEG and EMIMC/FMP DESs was increased [82,89]. However, SO_2_ solubility increased with increasing molar ratio of imidazole in ACC/imidazole DES because imidazole exhibited a strong interaction with SO_2_ [74]. A significant change in SO_2_ solubility was observed with the change in the molar ratio of ChCl and EMIMC-based DESs; however, that is not true for all DESs. For instance, betaine:EG and caprolactam:EG showed almost no change in SO_2_ absorption at different molar ratios (1:3 to 1:5) [49,76]. Similarly, a change in the molar ratio of PPZB/gly DES had almost no effect on the capacity of SO_2_ [94].

Yang et al. [106] investigated the effect of HBD of DESs [EMIMC/EG (1:1), EMIMC:TEG (1:1), and EMIMC:SNT (1:1)] on the solubility of SO_2_. The effect of HBD was ordered as SNT > EG > TEG for SO_2_ capture. The absorption capacity of SO_2_ by ChCl-based DESs was ordered as ChCl/thiourea (1:1) > ChCl/EG (1:2) > ChCl/malonic acid (1:1) > ChC/urea (1:2) at 0.1 MPa and 293.2 K [83]. Zhang et al. [92] prepared four DESs based on imidazole and its derivates to explore their performance with regard to SO_2_ capture. Imidazole, 2-methylimidazole, 2-ethylimidazole, and 2-propylimidazole were mixed with glycerol at a 1:2 molar ratio. Among the four DESs, the highest solubility of SO_2_ (0.253 g SO_2_/g DES at 0.002 MPa and 313.2 K) was achieved using imidazole/gly (1:2) DES [92]. Deng et al. [75] studied the solubility of SO_2_ in six DESs composed of quaternary ammonium salts (ChCl, ACC, TEAC, TEAB, TBAC, and TBAB) as the HBA and LV as the HBD at a 1:3 (HBA:HBD) molar ratio.

**Figure 8:**
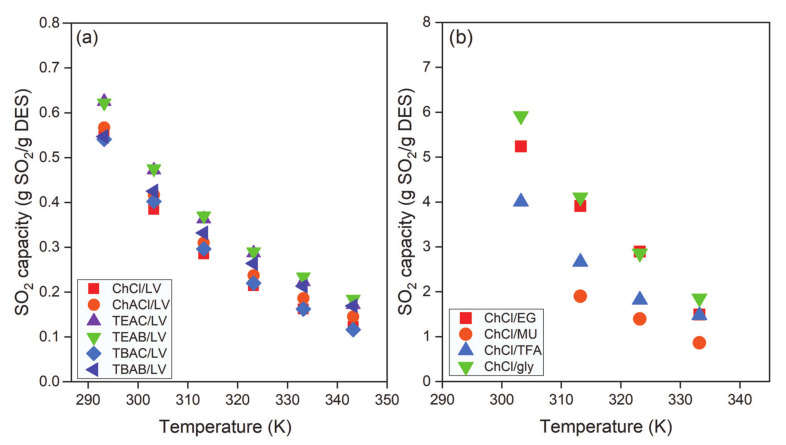
Effect of (**a**) HBA and (**b**) HBD on SO_2_ capacity at 0.1 MPa as a function of temperature. Data are extracted from [75,79].

The highest solubilities were obtained using TEAC/LV and TEAB/LV DES at all temperatures (293.2–343.2 K), as shown in Figure 8a. Long et al. [90] investigated the performance of four bisazole-based DESs in SO_2_ capture. The DESs were obtained by mixing EMIMC with imidazole, 1H-1,2,4-triazole, 1,2,3-1H-triazole, or tetrazole in a 2:1 (HBA:HBD) molar ratio. The effect of HBDs on SO_2_ capacity was examined at 293.2 K and 0.1 MPa, as illustrated in Figure 8b. The effect of HBDs on the solubility of SO_2_ was evident. TBAC was mixed with imidazole, 4-methylimidazole, pyrazole, tetrazole, and benzimidazole at a 1:2 molar ratio. Equilibrium was reached in 10 min, and the absorption capacities of SO_2_ were as follows: 4-methylimidazole > imidazole > benzimidazole > pyrazole > tetrazole. For EMIMC-based DESs, the effect of HBD in DESs on SO_2_ capture at all temperatures and 0.1 MPa was as follows: EMIMC/imidazole > EMIMC/1H-1,2,4-triazole > EMIMC/1,2,3-1H-triazole > EMIMC/tetrazole. It is also evident from Figure 8a that temperature plays a significant role in the capture of SO_2_ by DESs. SO_2_ capture via DESs decreased linearly with increasing temperature. Yang et al. also reported that the absorption capacity of SO_2_ using EMIMC/EG, EMIMC/TEG, and EMIMC/SNT decreased with increasing temperature [104,105,106].

#### 3.1.2. NOx Capture

**Figure 9:**
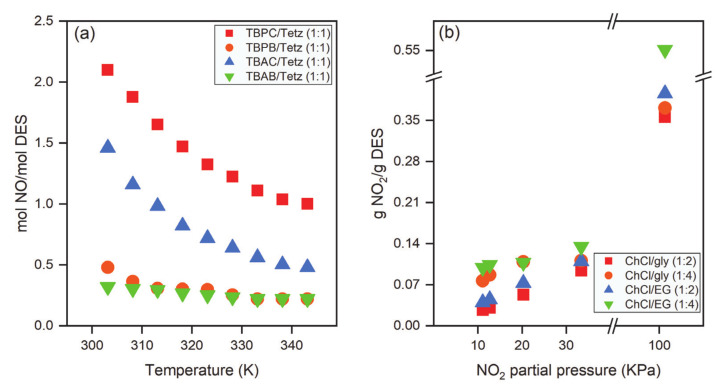
Effect of (**a**) temperature and HBA on the absorption capacity of NO, (**b**) partial pressure on _NO2_ solubilities in four ChCl-DESs at 298.15 K [81,84].

A limited number of studies have reported on the absorption of NO via DESs. Zhang et al. [92] used imidazole/gly (1:2) to capture NO. They used the same DES to capture SO_2_ and found that it had a very low capacity (0.034 mol NO/mol DES) for NO compared to SO_2_ (0.643 mol SO_2_/mol DES). Azole-based DESs were found to be efficient at dissolving NO. Zhang et al. [84] used four azole-based low-viscosity DESs to capture NO. TBPC/Tetz (1:1) exhibited the highest NO absorption capacity: 2.10 mol NO/mol DES at 0.1 MPa and 303.2 K. These researchers also studied the effect of temperature on the absorption capacity of NO, as shown in Figure 9a. The absorption capacity of NO decreased linearly with the increase in temperature. The authors found that there were chemical interactions between the hydrogen attached to the ring containing the nitrogen atom of Tetz and NO. The effect of HBA in DESs on the solubility of NO was also examined. The effect of HBA in DESs on NO capture at 0.1 MPa and 303.2 K was as follows: TBPC/Tetz > TBAC/Tetz > TBPB/Tetz > TBAB/Tetz. Moreover, lower NO pressure and higher temperature reduced the absorption capacity of the DESs. Sun et al. [107] explored the application of amine-based functional DESs for the capture of NO. The DESs were prepared by mixing ammonium salts with polyhydric alcohols, and all DESs showed good absorption capacity for NO (10 vol%). Sun et al. [107] also investigated the effect of temperature on the DES absorption capacity of NO. For example, the absorption capacity decreased from 0.33 to 0.18 mol NO/mol DES, with increasing temperature from 303.2 to 323.2 K in tetraethylenepentamine chloride (TEPA)/EG (1:3) DES. This could be attributed to the weak binding forces between NO and DES at higher temperatures. Owing to the increase in temperature, the equilibrium shifted in the opposite direction, causing reduction in the NO absorption capacity.

The effect of the molar ratio of DESs on the absorption capacity of NO was also investigated. For instance, NO absorption capacity increased from 3.10 to 4.52 mol NO/mol DES when the molar ratio of TEPA/EG changed from 1:1 to 1:3.

No change in the molar absorption capacity of NO was observed when the molar ratio further changed from 1:4 to 1:6. However, the mass absorption capacity of NO was reduced with the increase in EG from 1:4 to 1:6, which indicated that the active constituent affecting the NO absorption capacity was TEPA [107]. In another study, Sun et al. [96] reported that the absorption capacity of NO increased for TBPB/DMTU DES as the molar ratio increased from 1:1 to 1:3.

The effect of water on the absorption capacity of NO was also explored, and no significant change in the NO absorption capacity by TEPA/EG (1:3) DES was observed under different levels of water content [107]. For example, the mass absorption capacities of NO using TEPA/EG (1:3) DES were 0.30 and 0.32 mol NO/mol DES when the water content of the DES was 18.3 and 0.1 wt%, respectively. However, the mass transfer rate was improved with increasing water owing to a reduction in the viscosity of the DES.

Although applications of DESs for the capture of acid gases continue to emerge, DES absorption of NO_2_ is still in the embryonic stage. Recently, Chen et al. [81] studied the absorption of NO_2_ by ChCl-based DESs and their aqueous mixtures. The effect of the partial pressure of NO_2_ on the absorption capacity of DESs was analyzed at 298.15 K, as shown in Figure 9b. With decreasing NO_2_ partial pressure, the solubility of NO_2_ was reduced. For example, the solubility in ChCl/gly (1:2) was reduced from 0.356 to 0.027 gNO_2_/gDESs when the partial pressure was decreased from 101.3 to 0.01 MPa. The effect of the molar ratio of DESs on the absorption capacity was also investigated, and the solubility was increased with increasing molar ratio. The effect of water content on NO_2_ absorption capacity was also explored. With the addition of 3–6 wt% water, the solubility of NO_2_ in the DESs showed the following trend: ChCl/gly (1:2) > ChCl/EG (1:2) > ChCl/gly (1:4) > ChCl/EG (1:4). With high water content (more than 50 wt% water), NO_2_ absorption is unfavorable. At this water concentration, the DES becomes unstable, and a complex hydrogen bond network among the components of the DES and water is formed, as reported previously [81].

Other studies relevant to the absorption of NO_2_ in DESs were based on theoretical calculations alone. For instance, the absorption mechanism of NO_2_ in ChCl DESs with urea, methyl urea, and thiourea as HBD was investigated using quantum chemical methods [108]. Based on quantum calculations, ChCl/thiourea was found to be more favorable for denitrification. However, experimental data are needed to verify such predictions.

### 3.2. NH_3_ Capture

The excellent benefits of simple synthesis and high tunability have yielded a wide range of prospects for evolving DESs. Various configurations and combinations of HBA and HBD make DESs rich and diverse, affecting the physicochemical properties of these DESs and their ability to absorb NH_3_. This section focuses on the solubility of NH_3_ by various DESs. As illustrated in Table 3, NH_4_SCN, ChCl, and 1-ethyl-3-methylimidazolium ([emim]Cl) are the most widely used HBAs to absorb NH_3_. These HBAs are usually mixed with HBDs such as glycerol, EG, urea, benzoic acid, LV, and phenol to form DESs in a specific molar ratio.

**Figure 10:**
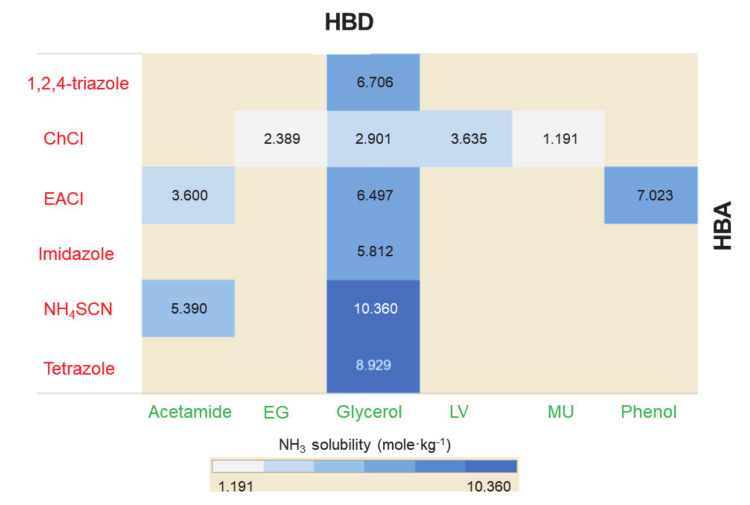
NH_3_ solubilities in DESs based on different HBAs and HBDs at 313.2 K and around 0.1 MPa. EACl/gly, 1,2,4-triazole/gly, imidazole/gly, tetrazole/gly were mixed in 1:3 HBA:HBD molar ratio. NH_4_SCN/gly and NH_4_SCN/urea were obtained by mixing in 2:3 molar ratio. All other DESs were prepared in 1:2 molar ratio [109,111,115,116,121].

The effect of the HBA and HBD of DESs is depicted by heat map induction, as shown in Figure 10. For the same molar ratio (1:2), ChCl-based DESs showed the following order for NH_3_ solubility: LV > gly > PNA > EG > TFA > urea > MU. During absorption by DESs, white solid particles were formed with the acidic HBDs (LV or PNA), which severely hindered NH_3_ interaction with the remaining absorbent and prevented further absorption. The breaking up of the supramolecular structure in the DES resulted in solid formation. This mechanism’s adverse consequence is well illustrated by the peculiar relationship between acidity and capacity in these solvents’ absorption.

Since carboxylic acids are even more acidic than alcohols and urea, DESs containing carboxylic acids as HBDs were assumed to have a higher NH_3_ uptake ability than ChCl/gly, ChCl/EG, and ChCl/urea. However, these predicted outcomes were not achieved [115]. This unexpected acidity–capacity relationship revealed that the well-recognized approach to increase the solubility of simple solutes by raising the acidity or their number of acidic groups of solvents is not generally true in the case of DESs because of the fragile supramolecular structure. In order to prevent this structural breakage, ternary DESs can be prepared by adding neutral donors. However, the opposite behavior was observed when acidic HBAs were used. For instance, Deng et al. [109] compared the performance of three azole-based DESs, and found that NH_3_ solubility increased with increasing HBA acidity as follows: tetrazole/gly > 1,2,4-triazole/gly > imidazole/gly. They also investigated the effect of HBD of DES on the solubility of NH_3_. Triazole was mixed with six different HBDs (caprolactam, acetamide, glycerol formal, DL-1,2-isopropylidene glycerol, EG, and glycerol). Higher solubility of NH_3_ was observed for the DESs containing a hydroxyl group as the HBD.

The addition of a third component to the binary DESs can affect the solubility of NH_3_. Zhong et al. [123] prepared a ternary DES by combining ChCl with EG and tetrazole and compared the solubility of NH_3_ of ternary DES with ChCl/EG and EG. ChCl/tetrazole/EG (3:7:14) showed greater absorption capacity compared to ChCl/EG (3:14) and EG. Moreover, ChCl/EG (3:14) exhibited the lowest NH_3_ solubility, demonstrating that the less-active component of NH_3_ solubility is ChCl. ChCl/tetrazole/EG (3:7:14) demonstrated higher NH_3_ capacities than EG at different pressures, indicating that tetrazole has the largest effect on NH_3_ capture. Furthermore, the capacities of NH_3_ in DESs were more pronounced at low pressures. For instance, using ChCl/tetrazole/EG (3:7:14), the solubility of NH_3_ was five times that in EG at 0.01 MPa and 313.2 K, while its solubility in ChCl/tetrazole/EG (3:7:14) was only 1.2 times that in EG at 0.1 MPa and 313.2 K.

**Figure 11:**
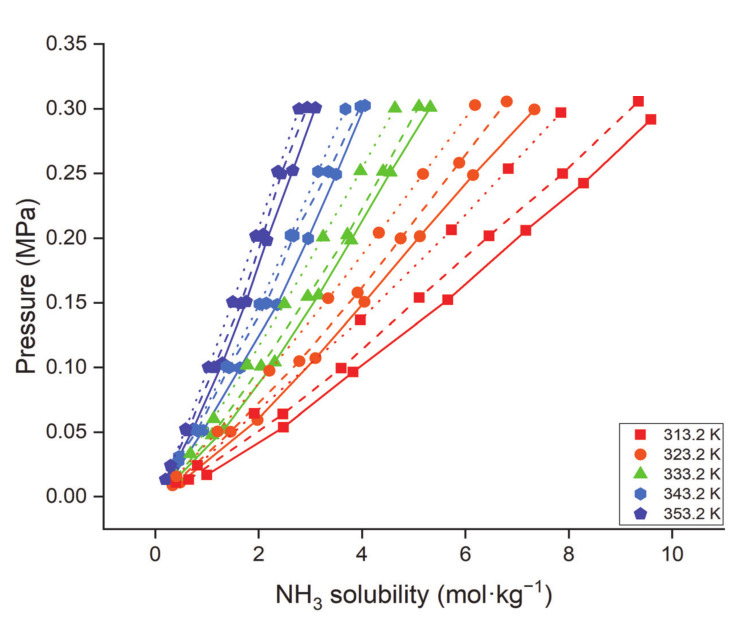
Effect of pressure, temperature, and molar ratio on the absorption capacity of NH_3_ through EACl/acetamide based DESs; solid, dash, and dot lines indicate the 1:1, 1:2, and 1:3 EACl/acetamide molar ratios, respectively. Data are taken from [117].

Figure 11 illustrates the effects of pressure, temperature, and DES molar ratio on the absorption capacity of NH_3_ [117]. NH_3_ absorption capacity increased with increasing pressure and decreasing temperature, which is a common behavior for gas absorption via liquids. However, for EACl-based DESs, as the temperature increased, the variation in NH_3_ solubility steadily faded, meaning that the strength of the hydrogen bond interactions between NH_3_ and EACl was negatively dependent on temperature. However, the isothermal profiles varied considerably from the ideal form, possibly because of the close interaction between EACl and NH_3_. EACl was thus the primary component in NH_3_ absorption mixtures. This assessment can also be concluded by comparing NH_3_ solubilities in EACl/acetamide DES mixtures with different EACl/acetamide molar ratios. EACl/acetamide 1:1 mixture had higher NH_3_ solubility owing to its higher EACl content compared to 1:2 and 1:3 molar ratios. A similar behavior was observed with EACl/urea-based DESs, i.e., the absorption capacities of NH_3_ decreased with increasing urea content in the EACl/urea mixtures [119]. However, for EACl/gly DESs, NH_3_ solubility decreased with increasing mole fraction of EACl in the DES. The order of capacity for EACl:gly DESs molar ratios was as follows: 1:5 > 1:4 > 1:3 > 1:2. For ternary DESs, there was no clear trend for NH_3_ solubility. For instance, the solubility increased (from 0.071 to 0.096 gNH_3_/gDES) when the ChCl/PNA/gly DES molar ratio increased from 1:3:1 to 1:3:3. However, the solubility was reduced (from 0.096 to 0.095 gNH_3_/gDES) after the molar ratio was further increased from 1:3:3 to 1:3:5. Similarly, ChCl/resorcinol (RES)/gly absorption capacity increased from 1:1:5 to 1:7:5 and then dropped sharply when the molar ratio was further changed to 1:9:5 [115]. This indicates that optimal mole fractions of DESs (either binary or ternary) are needed to enhance gas absorption. Furthermore, higher NH_3_ solubilities were observed at lower temperatures for EACl/acetamide, EACl/urea, and EACl/gly DESs.

The low viscosity characteristic of any solvent is favorable for the liquid transport and mass transfer of gas. The NH_3_ absorption was swift (with the equilibrium time being shorter than 1.5 min) in the azole-based DESs owing to their lower viscosity (<30 mPa. s at 298.2 K) [123]. GI/acetamide (1:2) DES also exhibited fast NH_3_ absorption owing to its low viscosity (21.05 mPa. s at 298.2 K), and the NH_3_ pressure was drastically reduced to a constant within around 100 s [120]. NH_3_ absorption capacity to achieve equilibrium was less than 60 s in EACl/phenol DESs, mainly due to their relatively low viscosities [118]. EACl/phenol DESs were prepared in different molar ratios. However, there was no significant difference between the NH_3_ solubility because there were minimal viscosity changes. Similarly, ternary DESs ChCl:tetrazole:EG (3:7:14) also resulted in very fast NH_3_ absorption (equilibrium time was less than 1.5 min) due to the low viscosity of DES (27.4 mPa. s at 298.2 K) [123].

### 3.3. H_2_S Capture

Liu et al. [124] found that H_2_S absorption capacity increased with increasing molar ratio of ChCl/urea DES from 1:1.5 to 1:2.5 at a fixed temperature and pressure. Furthermore, the absorption capacity of H_2_S in ChCl/urea DESs was decreased linearly by increasing the temperature. Carboxylic acid-based DESs showed higher solubility compared to ChCl/urea DESs [125]. For instance, the absorption capacity of H_2_S in ChCl/urea (1:2) is 0.38 (mole H_2_S/kg DES) at 313.2 K and 0.2 MPa, while it is 0.70 (mole H_2_S/kg DES) in ChCl/propionic acid (PA) (1:2) at 298 K and 0.184 MPa. The solubility of H_2_S in carboxyl acid-based-DESs was ordered as follows: TBAB/PA (1:1) > TBAB/AC (1:1) > TBAB/formic acid (FA) (1:1) > ChCl/PA (1:2) > ChCl/AC (1:2) > ChCl/FA (1:2) at 298 K and around 0.5 MPa. The effect of HBA on the solubility of H_2_S was also evident; i.e., for the same HBD, TBAB-based DESs showed higher H_2_S solubility compared to ChCl-based DESs. The hydrogen bond strength of TBAB-based DESs is lower than that of the ChCl-based DESs, and ChCl consists of a hydroxyl group; therefore, the resulting hydrogen bonding interactions in ChCl-based DESs are more complex than those in TBAB-based DESs. Recently, supported DESs using fumed silica (supporting material) and DES as the loading substance were developed to capture H_2_S [126]. Triethylamine hydrochloride (TEAC) and cupric chloride (CuCl_2_) were mixed at a 1:1 molar ratio to prepare the DES. The highest H_2_S capacity of 9.97 mg/g DES was obtained at a 10% DES loading rate and 303.2 K. Furthermore, it was also found that TEAC/CuCl_2_ (1:1) was more efficient as a loading substance than pure TEAC or CuCl_2_.

## 4. Selectivity of DESs in Capturing Gases

SO_2_ and CO_2_ are two typical gases that coexist in the flue gas. Thus, it is more important to selectively capture SO_2_ from simulated mixed gases containing both SO_2_ and CO_2_. Deng et al. [75] determined the SO_2_/CO_2_ selectivity (S) in six LV based DESs and compared the results with some ILs. Higher SO_2_/CO_2_ selectivities (134–199) were obtained using all six DESs than in the ILs (3–4 times higher). Therefore, LV-based DESs could be used as efficient absorbents for the selective capture of SO_2_ from CO_2_ in flue gas. Liu et al. [82] compared the performance of six phenol-based DESs for the selective absorption of SO_2_. Selectivity of SO_2_/CO_2_ using the phenol-based DES were as follows: ChCl/GC (1:3) > ChCl/GC (1:4) > ChCl/GC (1:5) > ChCl/cardanol (CD) (1:3) > ChCl/CD (1:4) > ChCl/CD (1:5) at 293.15 K and 0.1 MPa. SO_2_/CO_2_ selectivity (258) using ChCl/GC (1:3) was even higher than that of the LV-based DESs used by Deng et al. [75]. However, cardanol-based DESs had lower selectivity than GC-based DESs because the alkyl long chains in cardanol showed that weak interactions with SO_2_ and GC have clearly better capability to interact with SO_2_. Evidently, the increased molar ratio of phenols (GC or CD) to ChCl in DESs resulted in the decreased SO_2_/CO_2_ selectivity. The similar trend was observed for PPZB/gly DESs [94]. The order of selectivity in terms of PPZB/gly molar ratio was as follows: 1:4 (S = 33.1) > 1:5 (S = 12.8) > 1:6 (S = 9.5).

In industrial streams, other gases coexist with NH_3_, such as CO_2_ and N_2_. Therefore, it is imperative for solvents to capture NH_3_ as well as to exhibit higher selectivity for NH_3_ than for other gases. Li and co-authors [115] compared NH_3_/CO_2_ selectivity for binary and ternary DESs. ChCl/RES/gly (1:3:5) exhibited higher NH_3_/CO_2_ selectivity (142) than binary DES ChCl/RES (1:3) (64), indicating that the addition of glycerol to the binary DES did not increase the CO_2_ solubility but contributed to a much higher NH_3_ solubility. Furthermore, when resorcinol was replaced with phenol in ternary DES, lower selectivity (87) was recorded than in resorcinol-based ternary DES. When phenol was mixed with EACl in a 1:7 salt: alcohol molar ratio, better NH_3_/CO_2_ selectivities (S = 151–195 at 298.2–353.2 K) were demonstrated [118]. From the above discussion, it is obvious that glycerol as HBD and EACl as HBA play a significant role in the selective removal of NH_3_ from CO_2_. Therefore, EACl was mixed with glycerol at 1:2 molar ratio, and these exhibited excellent NH_3_/CO_2_ selectivities ranging from 818 to 5567 [116].

Azole-based binary and ternary DESs have also been investigated for the selective separation of NH_3_ from NH_3_/CO_2_ mixtures. The performance of 1,2,4-triazole/gly (1:3) and imidazole/gly (1:3) DESs was compared with some other DESs in term of selectivity. 1,24-triazole/gly (1:3) exhibited higher selectivity (216.3) than imidazole/gly (1:3), equivalent to that of ChCl/phenol/EG (1:5:4) DES (218), but lower than that of NH_4_SCN/gly (2:3) and ChCl/RES/gly (1:3:5) at 0.1 MPa and 313.2 K [113,115,121]. Azole-based ternary DES (ChCl/tetrazole/EG, 3:7:14) exhibited excellent selectivities ranging from 284–611 at 298.2–353.2 K and 0.043–0.1 MPa [123]. NH_3_/CO_2_ selectivity using EMIMC/1H-benzotriazole (1:2) was 198–107 at 298.2–353.2 K [127]. SO_2_/CO_2_ and NH_3_/CO_2_ selectivity data via DESs are collected in Table 4.

## 5. Desulfurization and Denitrification of Fuels

Because of limited available resources, oil refiners are presently processing crude oil with a higher content of sulfur and nitrogen compounds. The removal of sulfur and nitrogen is one of the major challenges in the fuel processing industry. Sulfur is usually found in fuels in the form of compounds such as thiophenes, mercaptans, and derivatives, which contribute to the emission of sulfur oxides during combustion. A number of studies on extraction desulphurization and denitrification using DESs have been reported to date. The extraction efficiency is dependent on the type of DESs used; therefore, the selection of suitable DESs is significant. Some important factors influencing the performance of DESs for sulfur and nitrogen removal include: molar ratio, type of HBA or HBD, and extraction temperature.

Desulfurization and denitrification are significantly affected by the HBA:HBD molar ratio. An increase in EG content in the DES results in higher extraction efficiency of nitrogen content. For example, ChCl/EG (1:3.5) exhibited higher extraction efficiency (70.9%) of pyridine compared to ChCl/EG (1:2) and ChCl/EG (1:3) [129]. Almashjary et al. [130] found that an increase in acid content in the DES increases the extraction efficiency of sulfur content. For instance, ChCl/PA exhibited higher extraction efficiency (~65%) of dibenzothiophene at 1:3 than at a 1:2 molar ratio in a single extraction stage. The extraction efficiency of thiophene, benzothiophene, and dibenzothiophene increased with increasing phenol content in the ChCl/phenol DESs. When the molar ratio of ChCl/phenol was increased from 1:2 to 1:4, the extraction efficiency increased; however, further increase in HBD content did not improve the extraction efficiency [131]. For TEAB/1,4-BD DES, the desulfurization efficiencies were reduced when the molar ratio was increased from 1:4 to 1:8 at the same temperature [132]. Sudhir et al. [133] studied the effect of molar ratio of phosphonium-based DESs on the extraction efficiency of dibenzothiophene. The extraction efficiency of dibenzothiophene using MTPPB/tetraethylene glycol (TetEG) DESs were as follows: MTPPB/TetEG (1:4) ~ MTPPB/TetEG (1:6) > MTPPB/TetEG (1:3). Both MTPPB/TetEG (1:4) and MTPPB/TetEG (1:6) exhibited almost equal extraction efficiency because it approached the saturation point of the desulfurization efficiency.

The sulfur removal efficiency is significantly dependent on the type of HBA and HBD. Li et al. [6] compared the performance of different HBA and HBDs in the DESs for the removal of sulfur. For the same HBD and molar ratio, the sequence for HBAs for sulfur removal efficiency was as follows: TBAC > TMAC > ChCl. The alkyl ammonium chloride-based DESs exhibited higher removal efficiency for benzothiophene than ChCl-based DES. The extraction sequence for HBDs was as follows: polyethylene glycol > propionate > EG > TEG > glycerol > malonic acid. Among HBDs, polyol-based DESs depicted higher extraction efficiency [extraction efficiency up to 71.06% using TBAC/PEG (1:2)]. Using acids as HBDS, the desulfurization efficiencies of some DES HBDs were as follows: *p*-toluenesulfonic acid (PTSA) > 5-sulfosalicylic acid > 4-aminosalicylic acid. A positive correlation was observed between DESs acidity and desulfurization efficiencies; i.e., DES with stronger acidity exhibits higher desulfurization capabilities [134]. Li and co-authors [135] also investigated the effect of different acidic HBDs of DES on the extraction of sulfur compounds. DESs were prepared by mixing acidic HBD with TBAB. The extraction capacity of different sulfur compounds was as follows: FA ~ AC ~ PA > oxalic acid (OA) > MA > adipic acid (AD) for thiophene; FA > PA > AC > OA > MA > AD for benzothiophene; PA > AC > OA > FA > MA > AD for dibenzothiophene. Regarding the nitrogen compound, a maximum removal efficiency of around 98.2% of carbazole was reported using ChCl/PNA (1:2) DES, while ChCl/MA (1:1) showed a very low efficiency of around 34.6% [7].

Concerning extraction temperature, studies [136,137,138] reported that the extraction efficiencies of sulfur content are reduced with increasing extraction temperature. A high temperature range was found to be unfavorable for the extraction of sulfur compounds using DESs. Warrag et al. [139] reported that the extraction temperature had a slight effect on the extraction efficiency of thiophene, attaining a maximum value of 30% at 313.2 K. Jha et al. [140] reported that the extraction efficiency of sulfur compounds was slightly increased with the increase in extraction temperature using diglycol-based DESs. Makoś and Boczkaj [131] studied the effect of extraction temperature on the extraction efficiency. The extraction efficiencies were found to increase with the increase in temperature from 293.2 K to 313.2 K; however, a further increase in temperature (313.2 K to 343.2 K) resulted in the reduction of extraction efficiency. Using TBAC/propionate (1:2) and TBAC/PEG (1:2) DESs, high extraction efficiency of around 71% of sulfur content was achieved, and equilibrium was reached in only 10 min. The viscosity of a solvent plays an important role in reaching equilibrium; i.e., a shorter equilibrium time would be achieved with lower solvent viscosity and higher extraction capability.

**Figure 12:**
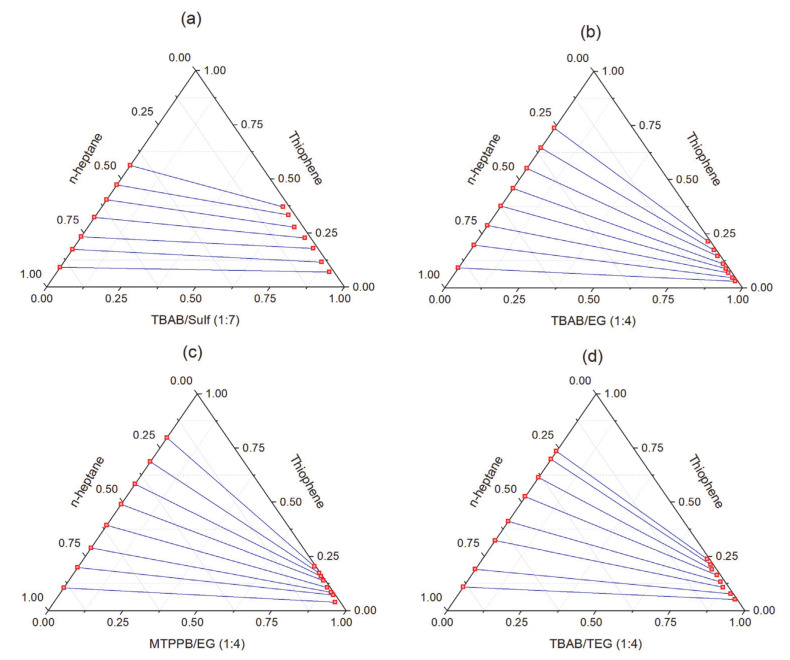
Experimental tie-lines for the ternary systems (**a**) thiophene + *n*-heptane + TBAB/Sulf (1:7), (**b**) thiophene + *n*-heptane + TBAB/EG (1:4), (**c**) thiophene + *n*-heptane + MTPPB/EG (1:4), and (**d**) thiophene + *n*-heptane + TBAB/TEG (1:4) at *T* = 298.15 K and 0.1 MPa. Data are taken from [143].

The LLE method has been widely applied for the removal of sulfur and nitrogen compounds. Hizaddin et al. [141] screened 94 DESs for potential applications in the extractive denitrification of diesel via a conductor-like screening model (COSMO-RS). The extraction efficiency of nitrogen compounds was investigated in terms of capacity, selectivity, and performance index. The screening results showed higher selectivity using ammonium-based DESs but higher capacity using phosphonium-based DESs. Moreover, DESs with amide and alcohols as HBDs resulted in higher selectivity, while DESs with carboxylic acid as the HBD exhibited higher capacity. The effect of molar ratio on the selectivity and capacity was not significant. In another study, the same group [142] compared the performance of two ammonium- and phosphonium-based DESs for the extraction of pyridine, pyrrole, indoline, and quinoline from *n*-hexadecane. Phosphonium based DES (TBPB/EG, 1:2) was found to have higher selectivity values and distribution ratio than ammonium-based DES toward nitrogen compounds. Hadj-Kali et al. [143] compared the performance of four DESs for the removal of sulfur compounds (thiophene) from *n*-heptane. The four systems are compared in a ternary diagram, as shown in Figure 12. The DES based on sulfolane (Sulf) as an HBD (TBAB/Sulf, 1:7) showed higher extraction efficiency of up to 35%.

The extraction efficiency of sulfur compounds depends on the alkyl-chain length of the HBA; for example, the DES with longer alkyl chain length on the HBA had a higher thiophene distribution capacity. Warrag et al. [128] found very high selectivity (higher than that of ILs and DESs) for thiophene using TEAC/gly (1:2) DES but the distribution capacity was lower using the same glycerol but DESs. When glycerol was replaced with EG in the DES, higher distribution coefficient was achieved compared to glycerol-based DES owing to the higher thiophene solubility in the EG-based DES. Alli and Kroon [8] studied the LLE of a compound consisting of both sulfur and nitrogen compounds (benzothiazole) via tetrahexylammonium bromide (THAB)-based DESs. THAB/EG (1:2) exhibited a higher (greater than unity) selectivity and distribution ratio for the extraction of benzothiazole from *n*-heptane. THAB/EG (1:2) was also used for the removal of a sulfur compound (thiophene) from *n*-hexane and *n*-octane. Both selectivity and distribution ratio were lower for thiophene (compared to benzothiazole) [127]. Various DESs used for the removal of sulfur and nitrogen compounds, along with their selectivity and distribution ratio, are presented in Table 5.

## 6. Conclusions

In this study, the use of DESs as green solvents for the capture of CO_2_, SOx, NOx, and NH_3_ gases was critically reviewed. We found that both components of the DESs play an important role in determining the solubility of these gases in the reported DESs. As expected, an increase in pressure and decrease in temperature increased the solubility of the gases in the DESs. However, the magnitude of enhancement varied depending on the type of components in the DESs. The highest CO_2_ absorption was in the amine-based DESs with a maximum value of 2.7 mol·kg^−1^ for ChCl/MEA (1:7) exceeding that in aqueous MEA. However, the solubility of CO_2_ depended strongly on the HBA and the range was from 0.338–2.700 mol·kg^−1^. In addition, it was found that both physical and chemical absorption of CO_2_ contribute to the solubility in amine-based DESs. The physical absorption will reduce the regeneration energy significantly. It is worth noting that not all amine based DESs had high CO_2_ solubility. The presence of water in the DESs affected the solubility of CO_2_ in most of the cases. Hence, it is of great importance to report the water content in DESs. Unfortunately, this was not done in many publications. It was found that the solubility of SO_2_ in DESs is comparable to that in ILs with a maximum of 1.54 g SO_2_/g DES for KSCN/caprolactam (1:3). For NO, the maximum solubility was 4.1 mol NO/mol DES for TBPC/DMTU (1:3) at 0.1 MPa and 303.2 K. The data for the solubility of NO_2_ in DES was scarce. The maximum solubility of NO_2_ was 0.551 mol NO_2_/mol DES at 298.2 K in ChCl/EG (1:2). Several DESs gave promising results for the absorption of NH_3_. The solubility of NH_3_ in ChCl/tetrazole/EG (3:7:14), ChCl/LV (1:4), and EaCl/phenol (1:7) was 9.952, 9.494, and 9.801 mol·kg^−1^, respectively. In all cases, the presence of water affected the solubility of gases in the DESs. It could be easily noted that a great effort is still needed to find a DES that can be used for the absorption of all acid gases discussed in this review.

Some reports indicated that the molar ratio of the components of the DES affected its ability to absorb the gases; however, other reports indicated that there are no significant changes in solubility with changes in the molar ratio. This could be attributed to the different chemical structure of the components of the DESs. Several methods, e.g., Redlich-Kwong and Peng-Robinson equation of state, were used to correlate the experimental data, and good agreement between the calculated and experimental results was achieved in most cases. Values of Henry’s law constant were calculated and reported for several DESs. In addition, COSMO-RS was used to predict the solubility of CO_2_ in some DESs. One important factor that was not given proper attention in the reported studies is the regeneration energy that is needed for the release of the dissolved gas from the DES. Moreover, the effect of the presence of more than one gas in the feed on the separation process must be investigated. However, it is clear that, except in the case of CO_2_, more work is still needed to understand the effect of different parameters on the solubility of SOx, NOx, and NH_3_ gases in DESs. In addition, more robust thermodynamic models for both correlating and predicting the solubility of these gases in different DESs must be tested. For CO_2_, pilot plant experiments should be performed in order to move toward commercial utilization of the selected DESs in the capture of CO_2_ under different operating conditions. In a parallel of this step, simulations using commercially available packages should be used to determine the optimum operational conditions of the process.

## Figures and Tables

**Table 1:** Solubility (*m*_CO2_) of CO_2_ in DESs at different temperatures and pressure.

DES	Molar Ratio	*T*, K	*P*, MPa	*m*_CO2_, mol·kg^−1^	Refs.
[bmim][MeSO_3_] ^1^/urea	1:1	303.15	0.423	0.245	[55]
ACC ^2^/1,2,4-triazole	1:1	303.15	0.497	0.186	[56]
ACC/guaiacol	1:3	303.15	0.432	0.127	[57]
	1:4	303.15	0.432	0.133	
	1:5	303.15	0.428	0.140	
ACC/imidazole	2:3	303.15	0.487	0.194	[56]
	1:2	303.15	0.526	0.239	
	1:3	303.15	0.479	0.249	
ACC/LV ^3^	1:3	303.15	0.543	0.301	[58]
Alanine/lactic acid	1:1	308.15	0.494	0.279	[59]
Alanine/ malic acid	1:1	308.15	0.493	0.346	
ATPPB ^4^/diethylene glycol	1:4	303.15	0.739	0.174	[60]
	1:10	303.15	0.734	0.145	
	1:16	303.15	0.742	0.122	
ATPPB/triethylene glycol	1:4	303.15	0.718	0.193	
	1:10	303.15	0.744	0.154	
	1:16	303.15	0.744	0.131	
Betaine/lactic acid	1:1	308.15	0.493	0.623	[59]
Betaine/malic acid	1:1	318.15	0.493	0.287	
BHDE ^5^/acetic acid	1:2	298.15	0.533	0.199	[61]
BHDE/lactic acid	1:2	298.15	0.866	0.122	
BTEA ^6^/acetic acid	1:2	298.15	0.551	0.265	
BTMA ^7^/acetic acid	1:2	298.15	0.530	0.271	
ChCl/MEA	1:7	298.15	0.651	2.700	
ChCl/guaiacol	1:3	303.15	0.434	0.116	[57]
	1:4	303.15	0.437	0.121	
	1:5	303.15	0.432	0.129	
ChCl/gly/acetic acid	1:1:1	298.15	0.542	0.112	[61]
DEH ^8^/guaiacol	1:3	303.15	0.428	0.153	[57]
	1:4	303.15	0.425	0.158	
	1:5	303.15	0.424	0.163	
GUA ^9^/MEA	1:2	298.15	0.563	0.827	[61]
MTOAB ^10^/decanoic acid	1:2	298.15	0.490	0.285	[62]
MTOAC ^11^/decanoic acid	1:2	298.15	0.490	0.297	
MTPPB ^12^/1,2-PD ^13^	1:4	298.15	0.861	0.228	[61]
MTPPB/acetic acid	1:4	298.15	0.652	0.390	
MTPPB/ethylene glycol	1:3	298.15	0.710	0.137	
MTPPB/gly	1:4	298.15	0.875	0.111	
MTPPB/LV	1:3	298.15	0.994	0.161	
MTPPB/LV/acetic acid	1:3:0.03	298.15	0.516	0.327	[61]
TBAB ^14^/acetic acid	1:2	298.15	0.715	0.380	
TBAB/MEA	1:6	298.15	0.654	1.036	
	1:7	298.15	0.637	1.208	
TBAB/LV	1:3	303.15	0.568	0.269	[58]
TBAB/octanoic acid	1:4	298.15	0.100	0.491	[63]
TBAB/PEG-8	1:4	298.15	0.100	0.286	
TBAC ^15^/acetic acid	1:2	298.15	0.631	0.393	[61]
TBAC/decanoic acid	1:2	298.15	0.490	0.337	[62]
TBAC/LV	1:3	303.15	0.559	0.303	[58]
TEAB ^16^/LV	1:3	303.15	0.564	0.240	
TEAC ^17^/acetic acid	1:2	298.15	0.530	0.284	[61]
	1:3	298.15	0.654	0.315	
TEAC/LV	1:3	303.15	0.562	0.274	[58]
TEAC/octanoic acid	1:3	298.15	0.624	0.342	[61]
TEMA ^18^/acetic acid	1:2	298.15	0.413	0.192	
TEMA/ethylene glycol	1:2	298.15	0.314	0.199	
TEMA/glycerol	1:2	298.15	0.833	0.126	
TEMA/lactic acid	1:2	298.15	0.418	0.109	
TEMA/LV	1:2	298.15	0.409	0.163	
TMAC ^19^/acetic acid	1:4	298.15	0.519	0.296	
TPAC ^20^/acetic acid	1:6	298.15	0.554	0.481	
TPAC/MEA	1:4	298.15	0.481	0.338	
	1:7	298.15	0.645	2.051	
TOAB ^21^/decanoic acid	1:2	298.15	0.490	0.288	[62]
TOAC ^22^/decanoic acid	1:1.5	298.15	0.490	0.305	
	1:2	298.15	0.490	0.307	

^1^ 1-butyl-3-methyl imidazolium methanesulfonate, ^2^ acetyl choline chloride, ^3^ levulinic acid, ^4^ allyltriphenylphosphonium bromide, ^5^ *N*-Benzyl-2-hydroxy-*N*,*N*-dimethyl ethanaminium chloride, ^6^ benzyltriethylammonium chloride, ^7^ benzyltrimethylammonium chloride, ^8^ diethylamine hydrochloride, ^9^ guanidinium hydrochloride, ^10^ methyltrioctylammonium bromide, ^11^ methyltrioctylammonium chloride, ^12^ methyltriphenyl phosphonium bromide, ^13^ 1,2 propanediol, ^14^ tetrabutylammonium bromide, ^15^ tetrabutylammonium chloride, ^16^ tetraethylammonium bromide, ^17^ tetraethylammonium chloride, ^18^ triethylmethylammonim chloride, ^19^ tetramethylammonium chloride, ^20^ tetrapropylammonium chloride, ^21^ tetraoctylammonium bromide, ^22^ tetraoctylammonium chloride.

**Table 2:** Solubility of SO_2_ (*m*_SO2_, g SO_2_/g DES), NO (*m*_NO_, mol NO/mol DES), and NO_2_ (*m*_NO2_, g NO_2_/g DES) in DESs and ILs at 0.1 MPa.

DES	Molar Ratio	*m*_SO2_, (*T*, K)	*m*_NO_, (*T*, K)	*m*_NO2_, (*T*, K)	Refs.
Deep eutectic solvents					
ACC/1,2,4-triazole	1:1	0.227 (303.2) ^1^			[74]
ACC/imidazole	1:1.5	0.356 (303.2) ^1^			
ACC/LV	1:3	0.567 (293)			[75]
ACC/imidazole	1:2	0.989 (303.2)			[74]
	1:3	0.383 (303.2) ^1^			
Betaine/EG	1:3	0.366 (313.2)			[76]
BMIMB ^2^/acetamide	1:1	1.00 (303.2)			[77]
BMIMB/DMU ^3^	1:1	0.920 (293.2)			[78]
BMIMC/ethyleneurea	1:1	1.07 (293.2)			
BMIMC/acetamide	1:1	1.17 (303.2)			[77]
BMIMC ^4^/DMU	1:2	0.950 (293.2)			[78]
	1:1	1.04 (293.2)			
	2:1	1.14 (293.2)			
BMIMC/Mim ^5^	1:1	1.31 (293.2)			[79]
	1:2	1.42 (293.2)			
BMIMC/imidazole	2:1	1.32 (293.2)			
	1:1	1.29 (293.2)			
	1:2	1.24 (293.2)			
ChCl/gly	1:1	0.678 (293.2)			[80]
	1:2	0.482 (293.2)		0.356 (298.2)	[80,81]
	1:3	0.380 (293.2)			[80]
	1:4	0.320 (293.2)		0.371 (298.2)	[80,81]
ChCl/LV	1:3	0.557 (293.2)			[75]
ChCl/GC	1:3	0.528 (293.2)			[82]
	1:4	0.501 (293.2)			
	1:5	0.479 (293.2)			
ChCl/cardanol	1:3	0.196 (293.2)			
	1:4	0.170 (293.2)			
	1:5	0.149 (293.2)			
ChCl/EG	1:2	0.700 (293.2)		0.396 (298.2)	[81,83]
	1:4			0.551 (298.2)	[81]
ChCl/malonic acid	1:1	0.490 (293.2)			[83]
ChCl/urea	1:2	0.350 (293.2)			
ChCl/thiourea	1:1	0.880 (293.2)			
ChCl/tetrazolium	1:1	0.860 (343.2)			[84]
ChCl/triazole	1:1	0.670 (343.2)			
ChCl/imid	1:1	0.470 (343.2)			
Caprolactam/imidazole	1:1	1.66 (303.2)			[85]
Caprolactam/acetamide	1:1	0.988 (303.2)			
Carnitine/EG	1:3	0.365 (313.2)			[76]
EMIMB/ethyleneurea	1:1	0.910 (293.2)			[78]
EMIMC ^6^/DMU	1:1	1.14 (293.2)			
EMIMC/EG	2:1	1.15 (293.2)			[86]
	1:1	1.03 (293.2)			
	1:2	0.820 (293.2)			
EMIMC/TEG	1:1	0.910 (293.2)			[87]
	2:1	1.06 (293.2)			
	4:1	1.20 (293.2)			
	6:1	1.25 (293.2)			
EMIMC/succinonitrile	1:1	1.13 (293.2)			[88]
	1:2	0.960 (293.2)			
	1:4	0.790 (293.2)			
EMIMC/FMP ^7^	1:1	0.220 (293.2)			[89]
	1:2	0.162 (303.2)			
	2:1	0.245 (303.2)			
EMIMC/acetamide	1:1	1.25 (303.2)			[77]
	1:2	1.13 (303.2)			
	2:1	1.39 (303.2)			
EMIMC/imidazole	2:1	1.40 (293.2)			[90]
EMIMC/1,2,4-triazole	2:1	1.28 (293.2)			
EMIMC/1,2,3-triazole	2:1	1.18 (293.2)			
EMIMC/tetrazole	2:1	1.13 (293.2)			
EMIMC/EPB ^8^	1:1	1.29 (293.2)			[91]
	2:1	1.34 (293.2)			
	3:1	1.39 (293.2)			
EMIMC/ethyleneurea	1:1	1.14 (293.2)			[78]
HMIMC ^9^/acetamide	1:1	1.02 (303.2)			[77]
Imidazole/gly	1:2	0.163 (313.2)	0.034 (313.2)		[92]
KSCN ^10^/acetamide	1:3	1.43 (293.2)			[93]
KSCN/caprolactam	1:3	1.54 (293.2)			
NH4SCN ^11^/acetamide	1:3	1.37 (293.2)			
NH4SCN/caprolactam	1:3	1.47 (293.2)			
PPZB ^12^/gly	1:4	0.420 (293.2)			[94]
	1:5	0.380 (293.2)			
	1:6	0.350 (293.2)			
TBAB/caprolactam	1:1	0.747 (293.2)			[95]
	1:2	0.764 (293.2)			
	1:3	0.719 (293.2)			
	1:4	0.696 (293.2)			
TBAB/LV	1:3	0.547 (293.2)			[75]
TBAB/Tetz ^13^	1:1		0.320 (303.2)		[84]
TBAB/DMTU ^14^	1:1		1.00 (303.2)		[96]
TBAB/imidazole	1:2	0.910 (293.2)			[79]
TBAB/caprolactam	1:2		0.090 (343.2)		[97]
TBAC/Mim	1:2	1.04 (293.2)			[79]
TBAC/imidazole	1:2	0.960 (293.2)			
TBAC/benzimidazole	1:2	0.820 (293.2)			
TBAC/pyrazole	1:2	0.710 (293.2)			
TBAC/tetrazole	1:2	0.460 (293.2)			
TBAC/ethyleneurea	1:1	0.810 (293.2)			[78]
TBAC/LV	1:3	0.541 (293.2)			[75]
TBAC/Tetz	1:1		1.46 (303.2)		[84]
TBAC/DMU	1:1	0.830 (293.2)			[78]
TBAC/DMTU	1:1	0.830 (293.2)	2.05 (303.2)		[96]
TBAC/caprolactam	1:2		0.130 (343.2)		[97]
TBAF ^15^/caprolactam	1:2		0.160 (338.2)		
TBPB ^16^/Tetz	1:1		0.480 (303.2)		[84]
TBPB/DMTU	1:1		1.13 (303.2)		[96]
TBPB/DMU	1:1		0.660 (293.2)		
	1:2		0.920 (293.2)		
	1:3		1.17 (293.2)		
TBPC ^17^/Mim	1:2	1.04 (293.2)			[79]
TBPC/DMU	1:1	0.830 (293.2)			[78]
TBPC/ethyleneurea	1:1	0.810 (293.2)			
TBPC/Tetz	1:1		2.10 (303.2)		[84]
TBPC/Imid	1:1		0.160 (303.2)		
TBPC/triazole	1:1		0.710 (303.2)		
TBPC/DMTU	1:1		2.13 (303.2)		[96]
	1:2		3.18 (303.2)		
	1:3		4.25 (303.2)		
TEAB/LV	1:3	0.622 (293.2)			[84]
TEAC/LV	1:3	0.625 (293.2)			
Ionic Liquids					
[Emim][SCN]		1.13 (293.2)			[98]
[NEt_2_C_2_Py][SCN]		1.06 (293.2)			[99]
[E_3_Py][Cl]		1.05 (293.2)			[100]
[E_3_Eim_2_][Cl]_2_		1.03 (293.2)			[101]
[Et_2_NEMim][Tetz]		1.10 (293.2)			[102]
[C_4_Py][SCN]		0.841 (293.2)			[103]

^1^ at 0.01 MPa, ^2^ 1-butyl-3-methylimidazolium bromide, ^3^ 1,3-dimethylurea, ^4^ 1-butyl-3-methylimidazolium chloride, ^5^ 4-methylimidazole, ^6^ 1-ethyl-3-methylimidazolium chloride, ^7^ *N*-formylmorpholine, ^8^ *N*-ethylpyridinium bromide, ^9^ 1-Hexyl-3-methyl-imidazolium chloride, ^10^ potassium thiocyanate, ^11^ ammonium thiocyanate, ^12^ 1- hydroxyethyl-1,4-dimethyl-piperazinium bromide, ^13^ tetrazolium, ^14^ 1,3-dimethylthiourea, ^15^ tetrabutyl ammonium fluoride, ^16^ tetrabutyl phosphonium bromide, ^17^ tetrabutyl phosphonium chloride.

**Table 3:** The solubility of NH_3_ (*m*_NH3_, mol·kg^−1^) in DESs at different temperature (K) and pressure (MPa).

DES	Molar Ratio	Temperature	Pressure	*m*_NH3_	Refs.
1,2,4-triazole/gly	1:3	313.15	0.10	6.706	[109]
ChCl/1,4-BD ^1^	1:3	313.15	0.13	2.347	[110]
	1:4	313.15	0.12	2.369	
ChCl/2,3-BD ^2^	1:3	313.15	0.13	2.073	
	1:4	313.15	0.12	1.903	
ChCl/1,3-PD ^3^	1:3	313.15	0.13	2.513	
	1:4	313.15	0.12	2.517	
ChCl/EG	1:2	333.2	0.10	1.491	[111]
ChCl/gly	1:2	333.2	0.11	1.341	
ChCl/MU ^4^	1:2	333.2	0.09	0.519	
ChCl/xylose	1:1	333.2	0.11	4.187	[112]
	1.5:1	333.2	0.11	3.722	
	2:1	333.2	0.10	2.980	
ChCl/TFA ^5^	1:2	333.2	0.12	1.475	[111]
ChCl/phenol/EG	1:5:4	313.2	0.10	6.988	[113]
	1:7:4	313.2	0.10	7.652	
ChCl/imidazole/EG	3:7:14	313.2	0.10	4.909	[106]
ChCl/triazole/EG	3:7:14	313.2	0.10	6.495	
ChCl/tetrazole/EG	3:7:14	313.2	0.11	9.952	
ChCl/urea	1:1.5	313.2	0.11	1.436	[114]
	1:2	313.2	0.11	1.599	
	1:2.5	313.2	0.10	1.355	
ChCl/PNA ^6^	1:2	313.2	0.10	2.445	[115]
ChCl/LV	1:2	313.2	0.10	4.631	
	1:4	298.2	0.10	9.494	[116]
	1:5	298.2	0.10	9.443	
EACl/AA ^7^	1:1	313.2	0.10	3.830	[117]
	1:2	313.2	0.10	3.600	
	1:3	323.2	0.10	2.210	
EaCl/phenol	1:2	313.2	0.10	7.023	[118]
	1:3	313.2	0.10	7.433	
	1:5	313.2	0.10	8.106	
	1:7	298.2	0.10	9.801	
EACl ^8^/gly	1:2	298.2	0.11	9.631	[116]
EACl/urea	1:0.5	313.2	0.10	4.396	[119]
	1:1	313.2	0.10	4.573	
	1:2	313.2	0.10	4.179	
GI ^9^/AA	1:2	303.15	0.10	5.300	[120]
	1:3	303.15	0.10	4.160	
	1:4	303.15	0.10	3.580	
Imidazole/gly	1:3	313.15	0.10	5.812	[109]
KSCN/gly	2:3	313.15	0.10	5.970	[121]
MAA ^10^/tetrazole	2:1	313.2	0.10	8.000	[122]
	2.5:1	313.2	0.10	6.650	
	3:1	313.2	0.10	5.940	
MAA/imidazole	2:1	313.2	0.11	1.770	
MAA/triazole	2:1	313.2	0.10	3.650	
NH_4_SCN/gly	2:3	313.15	0.10	10.36	[121]
NH_4_SCN/EG	1:3	313.15	0.10	9.890	
NH_4_SCN/urea	2:3	313.15	0.10	8.590	
NH_4_SCN/acetamide	2:3	313.15	0.10	5.390	
NH_4_SCN/caprolactam	1:3	313.15	0.10	1.730	
Tetrazole/gly	1:3	33.15	0.10	8.929	[109]

^1^ 1,4-butanediol, ^2^ 2,3-butanediol, ^3^ 1,3-propanediol, ^4^ *N*-Methyl urea, ^5^ trifluoroacetamide, ^6^ phenylacetic acid, ^7^ acetamide, ^8^ ethylamine hydrochloride, ^9^ guanidine isothiocyanate, ^10^ methylacetamide.

**Table 4:** Selectivity data of SO_2_/CO_2_ and NH_3_/CO_2_ using DESs.

DES	Molar Ratio	Selectivity	Refs.	DES	Molar Ratio	Selectivity	Refs.
SO_2_/CO_2_ selectivity at 293.15 K, 0.1 MPa				NH_3_/CO_2_ selectivity at 313.15 K, 0.1 MPa			
ACC/LV	1:3	155	[75]	1,2,4-triazole/gly	1:3	216	[109]
ChCl/LV	1:3	155		[Im][NO3] ^1^/EG	1:3	139	[128]
ChCl/GC	1:3	258	[82]	ChCl/Res/Gly	1:3:5	142	[115]
	1:4	237		ChCl/Res	1:3	64	
	1:5	214		ChCl/phenol/Gly	1:3:5	87	
ChCl/CD	1:3	36.0		ChCl/phenol	1:3	54	
	1:4	30.0		ChCl/Res/EG	1:3:5	49	
	1:5	26.0		ChCl/urea	1:2	16.7	[114]
TEAB/LV	1:3	183	[75]	ChCl/1,4-BD	1:3	74.7	[110]
TEAC/LV	1:3	199			1:4	79.1	
TBAB/LV	1:3	134		ChCl/2,3-BD	1:3	65.5	
TBAC/LV	1:3	141			1:4	52.9	
PPZB/gly	1:4	33.1	[94]	GI/AA	1:2	151 ^2^	[120]
	1:5	12.8			1:3	116 ^2^	
	1:6	9.50			1:4	972 ^2^	
				Imidazole/gly	1:3	37.3	[109]
				NH_4_SCN/gly	2:3	609	

^1^ imidazolium nitrate, ^2^ at 303.15 K.

**Table 5:** Selectivity and distribution ratio (D) for the removal of sulfur and nitrogen compounds using DESs.

DES	Mixture	S Range	D Range	Refs.
MTPPB/EG (1:4)	Benzothiazole/*n*-hexane	9.60–40.0	2.10–3.10	[144]
MTPPB/EG (1:4)	Benzothiazole/*n*-heptane	7.00–46.8	2.10–3.50	
THAB/EG (1:2)		7.00–27.50	2.36–3.76	[8]
THAB/gly (1:2)		7.33–35.26	1.90–2.99	
TBPB/EG (1:2)	Indoline/*n*-hexadecane	457–1,116	5.04–7.42	[142]
TBAB/EG (1:2)		833–2506	4.54–7.57	
MTPPB/gly (1:4)	Pyridine/*n*-hexane	26.1–839.5	1.589–2.677	[145]
MTPPB/EG (1:4)		34.1–232.6	2.50–2.60	[144]
MTPPB/EG (1:4)	Pyridine/*n*-heptane	30.1–276.9	2.60–3.40	
TBAB/EG (1:2)	Pyridine/*n*-hexadecane	727–1228	2.93–4.39	[142]
TBPB/EG (1:2)		157–437	3.24–4.60	
Betaine/LV (1:7)	Pyrene/*n*-decane	255.3–48,500	6.127–97.00	[146]
TBAB/EG (1:2)	Pyrrole/*n*-hexadecane	6659–46,953	30.31–94.00	[142]
TBPB/EG (1:2)		1413–8159	27.37–98.00	
MTPPB/TEG (1:4)	Quinoline/*n*-heptane	235.8–2327.9	4.120–9.574	[139]
TBPB/PTSA (1:1)		8644–10,866	363–467	[147]
TBPB/PTSA (1:1)	Quinoline/*n*-pentadecane	3740–24,321	200–277	
TBAB/EG (1:2)	Quinoline/*n*-hexadecane	3229–4955	3.56–5.00	[142]
TBPB/EG (1:2)		141–594	3.71–7.80	
TBAB/EG (1:4)	Thiophene/*n*-heptane	8.79–30.22	0.233–0.325	[143]
MTPPB/EG (1:4)		10.12–20.19	0.251–0.373	
TBAB/TEG (1:4)		10.70–51.95	0.302–0.464	
TBAB/Sulf (1:7)		13.77–41.87	0.659–0.764	
MTPPB/TEG (1:4)		20.80–161.40	0.402–0.647	[139]
THAB/EG (1:2)		1.89–9.55	0.81–0.95	[8]
THAB/gly (1:2)		3.21–14.8	0.66–0.79	
THAB/EG (1:2)	Thiophene/*n*-hexane	1.73–8.05	0.810–0.935	[148]
THAB/EG (1:2)		2.08–11.71	0.797–0.981	
TEAC/EG (1:2)		4.81–87.99	0.320–0.512	[149]
TEAC/gly (1:2)		42.68–257.73	0.130–0.226	
MTPPB/EG (1:3)		12.36–140.38	0.419–0.521	
MTPPB/gly (1:3)		1.42–29.30	0.031–0.158	
THAB/gly (1:2)	Thiophene/*n*-octane	1.40–12.99	0.662–0.789	[148]
THAB/gly (1:2)		1.66–17.30	0.665–0.794	
Betaine/LV (1:7)		14.4–159.7	0.396–0.489	[146]
TEAC/EG (1:2)		46.95–1009.11	0.378–0.701	[150]
TEAC/gly (1:2)		137.09–794.21	0.205–0.360	
MTPPB/EG (1:3)		26.44–465.49	0.409–0.550	
MTPPB/gly (1:3)		107.89–673.64	0.129–0.245	
